# Dental Depot—A Change

**Published:** 1869-01

**Authors:** 


### DENTAL DEPOT—A CHANGE.

It will interest and perhaps surprise the profession to learn that our much esteemed friend and brother, Dr. George Watt, has, for the present, at least, given up the active practice of the professsion; but he has not gone so far that we can not reach him, for he is more accessible now than before. He and Dr. N. W. Williams, of Xenia, have purchased the Dental furnishing establishment so successfully carried on for the past few years by J. S. Walter & Co., and formerly by John T. Toland. This has always been a popular Dental Depot, and we are sure will be none the less so under the new management. The present proprietors are well known to the profession throughout the West, and have personal acquaintance with a large majority. Their intimate acquaintance with Dental materials, instruments and appliances, will enable them to meet most fully the wants of the profession; their determination is to keep all supplies of the very best qualities.

We very much regret to lose Dr. Watt even temporarily as a practitioner, but we are very glad he turned his face no farther from us. The science of our profession will lose little or nothing by this change, but perhaps gain; for the strong probability is his health will be much improved by the change. The name of the firm is George Watt & Co. George Watt stands at the wheel. T.

